# The Telephone Language Screener (TLS): standardization of a novel telephone-based screening test for language impairment

**DOI:** 10.1007/s10072-023-07149-1

**Published:** 2023-11-27

**Authors:** Edoardo Nicolò Aiello, Veronica Pucci, Lorenzo Diana, Alessia Corvaglia, Aida Niang, Silvia Mattiello, Alice Naomi Preti, Giorgia Durante, Adele Ravelli, Lucia Consonni, Carolina Guerra, Adriana Delli Ponti, Gaia Sangalli, Teresa Difonzo, Stefano Scarano, Laura Perucca, Stefano Zago, Ildebrando Appollonio, Sara Mondini, Nadia Bolognini

**Affiliations:** 1 PhD Program in Neuroscience, School of Medicine and Surgery, University of Milano-Bicocca, Monza, Italy; 2 Dipartimento di Filosofia, Pedagogia e Psicologia Applicata (FISPPA), University of Padova, SociologiaPadua, Italy; 3 Human Inspired Technology Research Centre (HIT), University of Padova, Padua, Italy; 4 Laboratory of Neuropsychology, IRCCS Istituto Auxologico Italiano, Milan, Italy; 5 Department of Biomedical and Clinical Sciences “Luigi Sacco”, University of Milan, Milan, Italy; 6 Department of Psychology, University of Milano-Bicocca, Milan, Italy; 7 Fondazione IRCCS Ca’ Granda Ospedale Maggiore Policlinico, University of Milano, Milan, Italy; 8 Department of Neurorehabilitation Sciences, IRCCS Istituto Auxologico Italiano, Milan, Italy; 9 Department of Biomedical Sciences for Health, University of Milan, Milan, Italy; 10 Neurology Section, School of Medicine and Surgery, University of Milano-Bicocca, Monza, Italy

**Keywords:** Telemedicine, Cognitive screening, Aphasia, Language, Tele-neuropsychology

## Abstract

**Background:**

This study aimed at developing and standardizing the Telephone Language Screener (TLS), a novel, disease-nonspecific, telephone-based screening test for language disorders.

**Methods:**

The TLS was developed in strict pursuance to the current psycholinguistic standards. It comprises nine tasks assessing phonological, lexical-semantic and morpho-syntactic components, as well as an extra Backward Digit Span task. The TLS was administered to 480 healthy participants (HPs), along with the Telephone-based Semantic Verbal Fluency (t-SVF) test and a Telephone-based Composite Language Index (TBCLI), as well as to 37 cerebrovascular/neurodegenerative patients—who also underwent the language subscale of the Telephone Interview for Cognitive Status (TICS-L). An HP subsample was also administered an in-person language battery. Construct validity, factorial structure, internal consistency, test–retest and inter-rater reliability were tested. Norms were derived via Equivalent Scores. The capability of the TLS to discriminate patients from HPs and to identify, among the patient cohort, those with a defective TICS-L, was also examined.

**Results:**

The TLS was underpinned by a mono-component structure and converged with the t-SVF (*p* < .001), the TBCLI (*p* < .001) and the in-person language battery (*p* = .002). It was internally consistent (McDonald’s ω = 0.67) and reliable between raters (ICC = 0.99) and at retest (ICC = 0.83). Age and education, but not sex, were predictors of TLS scores. The TLS optimally discriminated patients from HPs (AUC = 0.80) and successfully identified patients with an impaired TICS-L (AUC = 0.92). In patients, the TLS converged with TICS-L scores (*p* = 0.016).

**Discussion:**

The TLS is a valid, reliable, normed and clinically feasible telephone-based screener for language impairment.

**Supplementary Information:**

The online version contains supplementary material available at 10.1007/s10072-023-07149-1.

## Introduction

Telephone-based cognitive screening (TBCS) plays a pivotal role within both clinical practice and research addressed to brain disorders, as allowing, *via* a widespread, highly accessible and practicable *medium*, the reduction of geographical, logistic, economical, socio-demographic and organizational barriers that undermine the access to such health facilities, the continuity of care and the viability/accomplishment of epidemiological studies and decentralized clinical trials [1–6].

Whilst several TBCS tools have been developed for detecting global cognitive impairment [7, 8], no standardized TBCS tests that focus on language are available, especially in Italy [9]. Indeed, within the Italian scenario, previously standardized TBCS test tap either into global cognition, *i.e.* the Italian telephone-based Mini-Mental State Examination (Itel-MMSE) [10], the Telephone Interview for Cognitive Status (TICS) [11, 12], the Tele-Global Exam of Mental State (Tele-GEMS) [13] and the ALS Cognitive Behavioral Screen™-Phone Version [14] or on overall executive efficiency, *i.e.* the Telephone-based Frontal Assessment Battery [15].

However, language dysfunctions—either primary or secondary to extra-linguistic deficits [16]—are common to a variety of neurological disorders of different aetiologies including degenerative [17, 18], vascular [19], neoplastic [20], traumatic [21], infective [22] and demyelinating ones [23].

In the light of such a trans-diagnostic relevance of language disorders, as well as of their prognostic entailments [24], practitioners and clinical researchers would undoubtedly benefit from the availability of TBCS tests that specifically tap into language [25]. Such a stance has been highlighted by De Witte *et al.* [26], who explored the feasibility of a telephone-based language battery (*i.e.* the TeleLanguage) for monitoring neurosurgical patients over time. However, no full standardization was provided for the TeleLanguage [26].

Given the above premises, this study aimed at standardizing the Telephone Language Screener (TLS) [27]—a novel, disease-nonspecific, telephone-based test aimed at screening for language deficits in patients with suspected/confirmed brain pathologies. More specifically, the current report focused on (1) assessing its psychometrics, (2) deriving its norms in an Italian population sample, and (3) offering preliminary feasibility evidence in a heterogeneous cohort of patients with neurodegenerative and cerebrovascular diseases.

## Methods

### Participants

The normative sample consisted of 480 Italian healthy participants (HPs) aged ≥18 years and with no history of (1) neurological/psychiatric disorders, (2) active psychotropic medications, (3) uncompensated/severe medical-general conditions and (4) uncorrected hearing deficits. Sample stratification is shown in Supplementary Table 1. HPs were recruited through both authors’ personal acquaintances and advertising at the University of Milano-Bicocca and the University of Padova.

Thirty-seven patients with neurological conditions were consecutively recruited at two clinics in Northern Italy according to neurologist-posed diagnoses pursuant to current diagnostic criteria: (1) 13 with small vessel disease (SVD) [28]; (2) 10 with neurodegenerative diseases (NDD), including 6 with hypokinetic extra-pyramidal disorders (2 with Parkinson’s disease [29] and 4 with atypical parkinsonism [30]), 3 with Alzheimer’s disease [31] and 1 with Lewy body disease [32]; and (3) 14 with neuroradiologically confirmed, left ischemic/haemorrhagic stroke. Patients were excluded if presenting with (1) uncorrected hearing deficits, (2) system/organ failures or (3) severe behavioural alterations.

### Materials

#### Telephone Language Screener

As to its structure, overall task content and aims, the TLS has been originally inspired by the Screening for Aphasia in NeuroDegeneration (SAND) [33]. The TLS includes nine tasks that provide both component-specific and global measures of language. Their development, pursuant to rigorous psycholinguistic standards, is detailed within the Supplementary Material 1. Briefly, such a process entailed the following: (1) the identification of an initial pool of items that, based on the neurolinguistic literature, were likely to be relatively sensitive to language deficits; (2) the conduction of pilot studies in HPs that allowed to refine the initial item sets by selecting the most feasible items and (3) assessing the definite item sets for their psycholinguistic features. The record-form and manual of the TLS are available upon request to the corresponding authors.

The TLS is structured into the following tasks:

- Connected Speech (CS); this task—adapted from Wilson et al*.* [34], Arcara and Bambini [35] and Catricalà et al*.* [33]—allows collecting speech samples within a semi-structured, ecological interview. It is subdivided into 2 subtasks (CSa; CSb) according to how speech productions is elicited, since different elicitation modalities allow exploring to different extents language components engaged in connected speech [36]. The CSa requires the examinee to describe her/his morning routine (narrative elicitation) within 40 s; the CSb requires to verbalize how to brush one’s teeth (procedural elicitation) within 20 s, and is a mere oral version of the written description task from the SAND itself [33]. CS performance (CSa + CSb) is scored on two levels: base and advanced (optional), as in Catricalà et al*.* [33]. The base CS scoring allows for a qualitative report of deficits within the phonological, lexical-semantic and morpho-syntactic components, as well as of features regarding articulatory deficits, speech fluency, communicative failures of a dysexecutive/inattentive aetiology [37]. According to Catricalà et al*.* [33], information units (IU, *i.e.* the most relevant meaning-conveying elements) represent the primary outcome of the CS task (range, 0–11). The advanced, optional scoring system encompasses a report of features suggestive of dysarthria and apraxia of speech [38] and allows a quantitative analysis of phonological, lexical-semantic and morpho-syntactic deficits [34, 37];
- Spelling; this task—adapted from Luzzatti et al*.* [39]—allows assessing the phonological component within the productive modality, in particular, the integrity of the phonological structure of lexical entries, by requiring to spell out a list of ten words. Stimuli are words that are not characterized by a perfect one-to-one correspondence between sound and letter, hence being moderately sensitive to phonological deficits [40];
- Semantic Association (SA); this task—adapted from Luzzatti et al*.* [41]—allows assessing the receptive, semantic component by requiring the examinee to choose which word among two (a target and a distractor) is more semantically related to a probe word (*e.g.* “Is dog most commonly associated with cat or pear?”). According to Luzzatti et al*.* [41], 6 triplets belong to three categories based on the type of the semantic association, which can be selectively impaired [42]: categorical (*i.e.* words relating to the same concept), functional (*i.e.* word referring to the same action), and visual-encyclopaedic (*i.e.* associations inferred from both spatial/context proximity of objects and knowledge acquired in educational settings);
- Naming to Description of Nouns (NtD-N) and Verbs (NtD-V); these tasks—adapted from Crepaldi et al*.* [43] and Aiello et al*.* [44]—assess both productive and receptive lexical-semantic components by requiring to name a target object/action (six items for both -N and -V) of which a verbal description is delivered (*e.g.* NtD-N: “Something which grows in the vegetable garden and is used for sauce, has seeds and is red. What is it?”; NtD-V: “The action that people do with their mouths on candles on their birthday”). NtD-N and -V items fit different classes (living *vs.* non-living nouns and transitive *vs.* intransitive verbs, respectively) in the view of covering the main types of category-specific deficits [45, 46];
- Comprehension and Memory Load (CML); this task allows assessing lexical-semantic and morpho-syntactic components within the receptive modality by requiring the examinee to perform six separate commands of progressive syntactic complexity in a fashion similar to the renowned Token Test [47]. Required actions entail both motor and verbal responses audible by the examiner. Complexity parameters are (1) syntactic structure (coordination *vs.* subordination links, *e.g.* “tap once on the table and tell what the colour of snow is” *vs.* “if the dog is an animal, say the day that comes before Friday”, respectively) and (2) length (*i.e.* two- *vs.* three-step commands). The CML ranges 0—15, as command components can be failed either separately or in clusters. For instance, to the item “tap once on the table and tell what the color of snow is” (range, 0–3), the examinee might perform both actions correctly (2 points assigned), yet in the wrong order (1 error);
- Repetition of Words (RoW), Non-Words (RoNW) and Sentences (RoS): these tasks—adapted from Catricalà et al*.* [33]—allow assessing the phonological component within both receptive and productive modalities by requiring the examinee to repeat, one at the time, six words, five non-words (*i.e.* phonologically legal strings with no meaning) and three sentences. Including words and non-words in repetition tasks is relevant to assess the integrity of both lexical-semantic and phonological routes, respectively involved in processing words with and without semantic representations within the mental lexicon [48].

The TLS-Total score is equal to the sum of IU, Spelling, SA, NtD-N, NtD-V, CML, RoW, RoNW and RoS sub-scores (range, 0–68). Additionally, the TLS comprises an “extra” Backward Digit Span (BDS) task, whose score is however not included within the TLS-Total score. Such a task—adapted from Monaco *et al.* (2013)—has been included in order to take into account phonological working memory/verbal short-term memory deficits that may affect language performances [49]. According to Pasotti *et al.* [50], two outcomes are computed: the longest sequence recalled, reflecting working memory capacity (BDS-WM); and the total number of sequences correctly reported (BDS-T), reflecting sustained attention during task execution.

#### Other measures

To the aim of convergent validity testing in HPs, the following measures were employed:

- The Telephone-based Semantic Verbal Fluency (t-SVF) task included within the Telephone-based Verbal Fluency Battery (t-VFB) [51], which was administered to 266 HPs;
- A Telephone-Based Composite Language Index (TBCLI; range, 0–7) computed as the sum of the language items included within the Itel-MMSE [10]—*i.e.*, naming-to-description of an object (*N* = 1) and sentence repetition (*N* = 1) —and the Tele-GEMS [13]—*i.e.*, naming-to-description of objects (*N* = 4) and comprehension of a bi-phasic command (*N* = 1)—, which was administered to *N* = 200 HPs.

When standardizing a cognitive test that is intended to be administered remotely, it is pivotal to ascertain that it taps into the same construct(s) that in-person measures—which should not overlap with the target test itself—tap into [9, 52, 53]. Thus, to this aim, a subsample of 79 HPs underwent a set of standardized, in-person language tasks that mimicked each section of the TLS – *i.e.*, the In-Person Composite Language Index (IPCLI). The IPCLI – described in Supplementary Table 2 – yields from the sum of the following measures (*range*=0-68): the Spelling task from the Edinburgh Cognitive and Behavioural ALS Screen [54], the Noun- and Verb-Naming tasks from the Esame NeuroPsicologico per l’Afasia [55] and the Semantic Association, Repetition of Words, Repetition of Non-Words, Repetition of Sentences, Sentence Comprehension and Connected Speech tasks from the SAND [33].

Finally, to the aim of convergent validity testing in patients, the Language sub-scale of the TICS (TICS-L; *range*=1-8) [11, 12] was administered. Patients also underwent the Mini-Mental State Examination [56] for clinical purposes.

### Procedures

All participants first underwent a semi-structured interview for collecting demographic data and medical history, as well as an in-depth sound-check for ensuring a good quality of the call – whose protocol has been described elsewhere [15, 51]. When tested over the telephone, participants were at their home; in-person testing took place at the Institutions involved in the study. TBCS sessions lasted ≤45’, whilst in-person evaluations lasted ≈30’.

For test-retest and inter-rater reliability testing, 29 HPs were re-administered the TLS after 30 days from the baseline and 26 TLS record-forms were scored online by two examiners blinded to each other’s scoring, respectively.

HPs undergoing both the TLS and the IPCLI were either first tested over the telephone (*N*=37) and then in-person at a 48-h distance or vice-versa (*N*=42), in order to rule out carry-over effects.

Data were collected by either licensed neuropsychologists or neuropsychology trainees; all examiners underwent an *ad hoc* training performed by the corresponding author. Data collection started in March 2021 and ended in May 2022.

### Statistical analyses

Convergent validity against telephone-based measures (both in HPs and in patients), as well the convergence between the TLS and the IPCLI, were tested through Spearman’s coefficients, since the vast majority of measures did not meet linear model analyses (*i.e.* skewness and kurtosis values >|1| and >|3|, respectively) [57].

In HPs, test-retest and inter-rater reliability were assessed *via* intra-class correlations, whereas internal consistency and factorial structure *via* McDonald’ω and a Principal Component Analysis (PCA), respectively.

Norms were derived through the Equivalent Score (ES) method [58, 59]. The ES method first entails a stepwise regression-based step that allows adjusting raw scores for significant demographic predictors. Subsequently, outer and inner tolerance limits (oTL; iTL) are identified on ranked adjusted scores (ASs) to provide a non-parametric, interval estimate of cut-off values. ASs≤oTL are attributed an ES=0, *i.e.* an “impaired” performance, whereas ASs≥*Mdn* an ES=4, *i.e.* a “high-end normal” performance. ASs comprised between the oTL and the *Mdn* are then allotted into three further ability levels, whose thresholds are identified *via* a *z-*score-based approach: ES=1 → “borderline”; ES=2 → “low-end normal performance”; ES=3 → normal performance. ASs comprised between the oTL and the iTL fall under the ES=1 but cannot be inferentially judged as either below- or above-cut-off.

In HPs, a 2-paramter logistic (2-PL) Item Response Theory (IRT) model [60, 61] was run *via* the R 4.1.0 package *mirt* [62] in order to estimate item difficulty and discrimination values for each TLS item – except for BDS (which is a task not included within the TLS-Total) and IU ones (which, theoretically, are not closed-ranged items). According to Arifin and Yusoff [61], difficulty values ranging from −3 to +3 were addressed as typical (with values ≤−3 indexing an extremely easy item and those ≥+3 an extremely difficult items), whereas, as to discrimination, values ranging from 0.65 to 1.34 were addressed as indexing moderate discrimination, and those ≥1.35 and >1.7 as indexing high and extremely high discrimination, respectively.

Clinical usability was tested *via* receiver-operating characteristics (ROC) analyses by comparing (1) the whole clinical group and each clinical subgroup against the normative sample and (2) patients with an impaired performance on the TICS-L (*i.e.* ES = 0) to those with an above-cut-off performance on it (*i.e.* ES ≥ 1).

Analyses were run through SPSS 28 (IBM Corp., 2021), R 4.1.0 (R Core Team, 2012), and jamovi 1.6.23 (the jamovi project, 2022). The significance level was set at *α* = 0.05 and Bonferroni’s correction was applied to multiple comparisons. Missing data were excluded pairwise.

### Power analyses

Based on Hobart *et al.*’s [63] recommendations, the minimum sample size for reliability and validity analyses in HPs were set at *N*=20 and *N*=80, respectively.

A sample size of 100 was deemed as sufficient for the PCA according to the guidelines delivered by Kyriazos [64].

According to Baylor *et al.*’s [60] rule-of-thumb suggestions, 250 observations were deemed as adequate to run the 2-PL IRT model.

In accordance with previous normative studies [11, 14, 15, 51], the minimum sample size for ES-based norming was estimated, through the R package *pwr* [65] at *N*=193, by addressing *f*^2^=.075, 1-β = 0.90, α = 0.05 and *df*_numerator_ = 3 (three predictors).

For ROC analyses comparing clinical groups to the normative sample, according to Obuchowski [66] and through the R package *easyROC* [67], the minimum sample sizes were estimated at *N*=30 and *N*=6, respectively, by addressing a case-control allocation ratio of 5, AUC = 0.8, 1-β = 0.8 and α = 0.05 within a single-test ROC analysis. For ROC analyses comparing patients with a defective *vs.* a normal TICS-L score, the minimum sample sizes were set at *N* = 4 and *N* = 20, respectively, by addressing an allocation ratio of 5, AUC = 0.85, 1-β = 0.8 and α = 0.05 within a single-test ROC analysis.

## Results

Demographic and telephone-based cognitive measures of the normative sample are shown in Table 1. Supplementary Table 3 reports IPCLI measures of the target HC subsample. Ceiling effects in the TLS-Total, defined as a score ≥95th percentile of the normative performance, was detected in 6% of the sample.

**Table 1:** Demographic and telephone-based cognitive data of the normative sample

N	480
Age (years)	51.79 ± 18.61 (18–96)
Sex (male/female)	191/289
Education (years)	12.88 ± 3.96 (4–25)
Italian regions (%)	
North Italy	78%
Center Italy	7.7%
South Italy	14.3%
Occupation (%)	
Predominantly manual	25.7%
Manual/clerical	26.3%
Predominantly clerical	48%
Telephone-based Composite Language Index^a*^	6.67 ± .67 (3–7)
Telephone-based Semantic Verbal Fluency^b^	56.06 ± 13.34 (3–83)
Telephone Language Screener	
Total	61.13 ± 4.99 (34–68)
Informative Units	8.37 ± .1.90 (0–11)
Backward Digit Span-Total^c^	4.79 ± 2.04 (0–8)
Backward Digit Span-Working Memory^c^	4.68 ± 1.32 (0–6)
Spelling	8.92 ± 1.54 (0–10)
Semantic Association	5.87 ± .39 (3–6)
Naming to Description of Nouns	5.45 ± .87 (1–6)
Naming to Description of Verbs	5.53 ± .81 (2–6)
Repetition of Words	5.85 ± .20 (4–6)
Repetition of Non-Words	4.28 ± .94 (0–5)
Repetition of Sentences	2.72 ± .54 (1–3)
Comprehension and Memory Load	14.13 ± 1.81 (1–15)

^a^Data available for *N* = 200 participants

^b^Data available for *N* = 266 participants

^c^Data available for *N* = 401 participants

^*^Derived as the sum of the language items included within the Italian telephone-based Mini-Mental State Examination [10] and the Tele-Global Examination of Mental State[13]

At α_adjusted_= 0.025, the TLS-Total was associated with both the t-SVF (*r*_*s*_(266)= 0.33; *p* < .001) and the TBCLI (*r*_*s*_(201)= 0.25; *p* < .001). Moreover, a significant association was detected between the TLS-Total and the IPCLI (*r*_*s*_(79) = 0.34; *p* = .002).

The TLS was underpinned by a mono-component structure (29.38% of variance explained), with all tasks yielding substantial loadings (range, 0.47–0.70), except for RoW and IU tasks (< 0.3). Consistently, the TLS was acceptably reliable at an internal level (McDonald’ω = 0.67), with item-rest correlations ranging from 0.30 to 0.47, except for RoW (0.10) and IU tasks (0.05). High test-retest (ICC = 0.83) and inter-rater reliability (ICC = 0.99) was detected.

Table 2 shows item difficulty and discrimination values. The majority of TLS items fell within a typical difficulty range, albeit towards easiness, with only a limited number of them being classified as extremely easy. Spelling items proved to be the most difficult ones. As to discrimination, the vast majority of TLS items proved to come with moderate discriminative values, with some of those included within Spelling, SA, NtD-N, NtD-V, RoW and CML yielding high-to-extremely-high discrimination.

**Table 2:** Item difficulty and discrimination values as yielded by the 2-PL IRT model in HPs (*N* = 401)

Item	Difficulty	Discrimination
Spelling 1	− 2.09	1.77^●^
Spelling 2	− 1.88	2.11^●^
Spelling 3	− 1.55	2.68^●^
Spelling 4	− 1.78	1.97^●^
Spelling 5	− 2.37	.60
Spelling 6	− 2.03	1.42^▲^
Spelling 7	− 1.37	.97
Spelling 8	− 2.27	1.61^▲^
Spelling 9	− 2.21	1.85^●^
Spelling 10	− 2.82	1.10
Semantic Association 1	− 2.85	1.97^●^
Semantic Association 2	n.a	-.08
Semantic Association 3	− 3.68^■^	1.65^▲^
Semantic Association 4	− 4.02^■^	1.34
Semantic Association 5	− 3.71^■^	1.51^▲^
Semantic Association 6	− 2.62	1.60^▲^
Naming to Description of Nouns 1	− 2.35	1.26
Naming to Description of Nouns 2	− 2.99	.89
Naming to Description of Nouns 3	− 2.26	.98
Naming to Description of Nouns 4	− 2.46	1.51^▲^
Naming to Description of Nouns 5	− 8.76^■^	.31
Naming to Description of Nouns 6	− 1.93	1.15
Naming to Description of Verbs 1	− 2.30	1.19
Naming to Description of Verbs 2	− 2.70	1.19
Naming to Description of Verbs 3	− 3.41^■^	.87
Naming to Description of Verbs 4	− 6.17^■^	.72
Naming to Description of Verbs 5	− 2.06	1.39^▲^
Naming to Description of Verbs 6	− 1.47	1.14
Repetition of Words 1	− 3.59^■^	1.72^●^
Repetition of Words 2	n.a	.28
Repetition of Words 3	− 2.78	4.49^●^
Repetition of Words 4	− 5.59^■^	.95
Repetition of Words 5	− n.a	.07
Repetition of Words 6	− 8.98^■^	.35
Repetition of Non-Words 1	− 2.58	.71
Repetition of Non-Words 2	− 5.37^■^	.42
Repetition of Non-Words 3	− 1.80	.86
Repetition of Non-Words 4	− 2.15	.96
Repetition of Non-Words 5	− 7.78^■^	.33
Repetition of Sentences 1	− 5.97^■^	.82
Repetition of Sentences 2	− 1.50	1.26
Repetition of Sentences 3	− 2.33	1.27
Comprehension and Memory Load 1*	− 2.72	1.50^▲^
Comprehension and Memory Load 2*	− 3.51^■^	1.39^▲^
Comprehension and Memory Load 3*	− 4.23^■^	1.03
Comprehension and Memory Load 4*	− 2.40	3.69^●^
Comprehension and Memory Load 5*	− 3.45^■^	1.15
Comprehension and Memory Load 6*	− 3.14^■^	1.24

*n.a.* not applicable (the model yielded inadequate estimates), *TLS* Telephone Language Screener, *2-PL* two-parameter logistic, *IRT* Item Response Theory, *HPs* healthy participants

^*^Item not originally dichotomous and thus dichotomized based on a raw score above *vs.* below the 5th percentile of the empirical distribution

^■^Extremely easy item

^▲^High discrimination

^●^Extremely high discrimination

Table 3 shows adjustment equations for raw TLS measures as well as TLs and ES thresholds for TLS ASs. Norms were derived from the whole sample for all TLS tasks except for the advanced CS scoring measures, which were derived from *N*=219 HPs (see Supplementary Table 4 for the stratification of this sub-sample and Supplementary Table 5 for its descriptive statistics). Norms for the BDS-T/-WM were instead derived from *N*=401 HPs. An automated AS and ES calculation sheet is provided within the Supplementary Material 2. RoW and IU, as well as a number of advanced CS scoring measures, were not predicted by either age, education or sex. Age negatively predicted the vast majority of measures (*p*s < .05), at times concurrently with education (*p*s < .05), which instead was a positive predictor. No sex differences emerged as to all TLS measures (*p*s ≥ .06).

**Table 3:** Adjustment equations and Equivalent Score thresholds for raw TLS measures

	Adjustment equations						
Base measures^**a**^							
Total	AS = RS + 0.000009*[(age^3)-191,433.997917] + 50.82466*[(1/education)-0.088235]						
Informative units	n.a						
Spelling	AS = RS + 0.000001*[(age^3)-191,433.997917] + 16.71391*[(1/education)-0.088235]						
Semantic association	AS = RS + 2.153293*[(1/education)-0.088235]						
Naming to description of nouns	AS = RS + 0.000001*[(age^3)-191,433.997917] + 5.834264*[(1/education)-0.088235]						
Naming to description of verbs	AS = RS -0.206562*[ln(100-age)-3.787995] + 5.803237*[(1/education)-0.088235]						
Comprehension and memory load	AS = RS + 0.000003*[(age^3)-191,433.997917] + 17.26772*[(1/education)-0.088235]						
Repetition of words	n.a						
Repetition of non-words	AS = RS + 0.000002*[(age^3)-191,433.997917]						
Repetition of sentences	AS = RS + 0.0000005*[(age^3)-191,433.997917] + 2.697296*[(1/education)-0.088235]						
Backward Digit Span^**b**^							
Total	AS = RS + 0.000002*[(age^3)-193,861.925187] + 16.46647*[(1/education)-0.088601]						
Working memory	AS = RS + 0.000001*[(age^3)-193,861.925187] + 10.25621*[(1/education)-0.088601]						
Advanced CS scoring^**c**^							
Number of words	AS = RS-12.54032*[√(education)-3.575916]						
Number of sentences	AS = RS-2.644981*[ln(100-age)-3.786775]-5.560185*[log_10_(education)-1.095679]						
Nouns/words ratio	AS = RS + 0.000007*[(age^2)-3032.251142]						
Verbs/words ratio	AS = RS+ 0.00000004336*[(age^3)-188,419.666667]						
Function words/words ratio	n.a						
			Equivalent Scores				
Base measures^**a**^	oTL	iTL	0	1	2	3	4
Total	52.64	54.9	≤ 52.64	52.65–56.41	56.42–58.79	58.8–61.5	≥ 61.51
Informative units	3	6	≤ 3	4–7	8	9–10	11
Spelling	5.86	6.88	≤ 5.86	5.87–7.41	7.42–8.41	8.42–9.28	≥ 9.29
Semantic association	4.94	5.12	≤ 4.94	4.95–5.9	5.91–5.94	5.95–5.99	6
Naming to description of nouns	3.67	4.13	≤ 3.67	3.68–4.64	4.65–4.97	4.98–5.72	≥ 5.73
Naming to description of verbs	3.7	4	≤ 3.7	3.71–4.7	4.71–5.2	5.21–5.81	≥ 5.82
Comprehension and memory load	10.52	12.14	≤ 10.52	10.53–12.73	12.74–13.91	13.92–14.32	≥ 14.33
Repetition of words	4	6	≤ 4	-	-	-	-
Repetition of non-words	2.5	2.78	≤ 2.5	2.51–3.01	3.02–3.77	3.78–4.64	≥ 4.65
Repetition of sentences	1.41	1.89	≤ 1.41	1.42–1.96	1.97–2.82	2.83–2.9	≥ 2.91
Backward Digit Span^**b**^							
Total	1.20	1.92	≤ 1.20	1.21–2.50	2.51–3.53	3.54–4.78	≥ 4.79
Working memory	1.58	2.90	≤ 1.58	1.59–3.49	3.50–4.00	4.01–4.81	≥ 4.82
Advanced CS scoring^**c**^							
Number of words	51.14	57.63	≤ 51.14	51.15–58.68	58.69–72.37	72.38–88.80	≥ 88.81
Number of sentences	9.78	12.44	≤ 9.78	9.79–13.02	13.03–14.97	14.98–17.09	≥ 17.10
Nouns/words ratio	0.138	0.160	≤ 0.138	0.139–0.164	0.165–0.196	0.197–0.227	≥ 0.228
Verbs/words ratio	0.151	0.173	≤ 0.151	0.152–0.183	0.184–0.208	0.209–0.235	≥ 0.236
Function words/words ratio	0.306	0.338	≤ 0.306	0.307–0.347	0.348–0.377	0.378–0.404	≥ 0.405

*TLS* Telephone Language Screener, *AS* adjusted score, *CS* Connected Speech, *RS* raw score, *oTL* outer tolerance limit, *iTL* inner tolerance limit, *n.a.* adjustment equation not available (no significant demographic predictors for this measures)

^a^Norms computed on the whole sample (*N* = 480)

^b^Norms computed on *N* = 401 healthy participants

^c^Norms computed on *N* = 219 healthy participants

Table 4 shows patients’ background and cognitive data. The TLS was moderately-to-highly accurate in discriminating the normative sample from both the whole clinical group (AUC = 0.80; *SE* = 0.04; CI 95% [0.72, 0.87]) and each clinical subsample (SVD: AUC = 0.78; *SE*= 0.06; CI 95% [0.65, 0.90]; NDD: AUC = 0.84; *SE* = 0.06; CI 95% [0.72, 0.95]; left stroke: AUC = 0.79; *SE* = 0.06; CI 95% [0.67, 0.91]). Moreover, in identifying patients with a defective TICS-L (*N*=4), the TLS showed high accuracy (AUC = 0.92; *SE* = 0.05; CI 95% [0.83, 1]). Finally, within the whole patient group, the TICS-L was associated with the TLS (*r*_*s*_(36) = 0.40; *p* = .016).

**Table 4:** Patients’ background and cognitive data

	Total	SVD	NDD	Left stroke
*N*	37	13	10	14
Sex (male/female)	18/19	4/9	7/3	7/7
Age (years)	73.2 ± 12.84 (38–92)	80.31 ± 7.4 (62–91)	77.7 ± 4.42 (70–82)	63.29 ± 14.85 (38–92)
Education (years)	13.76 ± 4.37 (3–18)	11.54 ± 5.08(3–18)	15.4 ± 3.63 (8–18)	14.64 ± 3.48 (8–18)
MMSE (*N* = 34)^a^	27.75 ± 2.61 (20–30)	27.75 ± 2.53(22–30)	26.88 ± 3.23 (20–30)	28.63 ± 2 (25–30)
TICS (*N* = 36)^b^				
Total	31.44 ± 5.28 (17–41)	29.77 ± 5 (21–38)	31.11 ± 5.92 (17–38)	33.21 ± 4.9 (21–42)
TICS-L	7.39 ± .93 (4–8)	7.46 ± .78 (6–8)	7.33 ± 1.32 (4–8)	7.36 ± .84 (6–8)
TLS				
Total	52.49 ± 9.99 (28–64)	53.08 ± 9.91(35–64)	50.7 ± 12.08 (28–63)	53.21 ± 9.01 (38–64)
IU	7.19 ± 2.38 (3–11)	7.23 ± 2.45 (3–11)	7.6 ± 2.27 (4–11)	6.86 ± 2.51 (3–11)
BDS-T	3.65 ± 2.47 (0–8)	3 ± 3.13 (0–8)	3.7 ± 2.06 (1–6)	4.21 ± 2.04 (1–7)
BDS-WM	4.19 ± 1.33 (2–6)	3.69 ± 1.6 (2–6)	4.2 ± 1.03 (3–6)	4.64 ± 1.15 (3–6)
Spelling	6.97 ± 3.44 (0–10)	6.69 ± 3.5 (0–10)	6.3 ± 3.53 (0–10)	7.71 ± 3.43 (0–10)
SA	5.81 ± .4 (5–6)	5.69 ± .48 (5–6)	5.8 ± .42 (5–6)	5.93 ± .27 (5–6)
NtD-N	4.51 ± 1.48 (0–6)	4.54 ± 1.39 (2–6)	3.8 ± 1.87 (0–6)	5 ± 1.11 (3–6)
NtD-V	4.57 ± 1.41 (0–6)	4.69 ± 1.11 (3–6)	4.5 ± 1.43 (2–6)	4.5 ± 1.7 (0–6)
RoW	5.68 ± .53 (4–6)	5.69 ± .48 (5–6)	5.5 ± .71 (4–6)	5.79 ± .43 (5–6)
RoNW	3.38 ± 1.42 (0–5)	3.92 ± 1.19 (1–5)	3.3 ± 1.42 (0–5)	2.93 ± 1.54 (0–5)
RoS	2.27 ± .9 (0–3)	2.54 ± .78 (1–3)	2.1 ± .99 (0–3)	2.14 ± .95 (0–3)
CML	12.11 ± 3.13 (3–15)	12.08 ± 2.66 (7–15)	11.8 ± 3.88 (3–15)	12.36 ± 3.18 (4–15)

*SVD* small vessel disease, *NDD* neurodegenerative diseases, *MMSE* Mini-Mental State Examination, *TICS* Telephone Interview for Cognitive Status, *TICS-L* Telephone Interview for Cognitive Status-Language, *BDS-T* Backwards Digit Span-Total, *BDS-WM* Backwards Digit Span-Working Memory, *SA* Semantic Association, *NtD-N* Naming to Description of Nouns, *NtD-V* Naming to Description of Verbs, *RoW* Repetition of Words, *RoNW* Repetition of Non-Words, *RoS* Repetition of Sentences, *CML* Comprehension and Memory Load, *IU* Information Units

^a^Data available for *N* = 34 patients

^b^Data available for *N* = 36 patients

## Discussion

The present work provides Italian practitioners and researchers with a standardized, disease-nonspecific TBCS test for language impairment, *i.e.* the TLS [27], along with preliminary evidence on its clinical usability in neurodegenerative and cerebrovascular patients. The TLS adds up to the range of standardized TBCS tests that are currently available in Italy [10–15]—in order to improve tele-neuropsychological practice within both clinical and research settings [9]. Remarkably, the development and comprehensive standardization of a TBCS test for language impairment is unprecedented within the international literature; the procedures herewith described will thus hopefully stand as a virtuous paradigm for future research on this topic, as well as for adaptations of the TLS to other languages and cultures.

The TLS has been indeed developed according to rigorous psycholinguistic/neurolinguistic standards, proved to be valid (both at the structure and construct levels) and reliable (at an internal, test-retest and inter-rater level), as well as to converge with in-person language measures. Additionally, item-level information for the TLS has been herewith provided—with the aim of easing the interpretation of its results [9, 68]. Moreover, as coming with regression-based norms for both its total score and each of its tasks, the TLS allows detecting both overall and component-specific language deficits. In this respect, the inclusion of normed BDS tasks also allows for qualitatively determining whether phonological working memory/verbal short-term memory deficits impact on TLS scores. Specific sections have been then developed within the TLS record-form to qualitatively report relevant semeiotic elements related to motor speech disorders and overall communicative failures, as well as to quantify connected speech deficits—the latter aspect being of major relevance in the light of the promising role of speech sample analyses towards an early detection of cognitive decline in a variety of brain disorders [36].

The TLS was also shown to be able both to discriminate HPs from neurological patients and to identify, within a clinical cohort, the occurrence of language deficits (*i.e.* a defective TICS-L score) in cerebrovascular and neurodegenerative diseases. Similarly to the SAND [33], which represented a relevant source of inspiration for its development, the TLS should be thus intended as a disease-nonspecific language screener to be applied for case-findings aims whenever deemed as appropriate—*i.e.*, also beyond primary aphasic syndromes [69–71]. In this regard, it has to be nevertheless noted that the SAND has been specifically intended to be administered to patients with neurodegenerative disorders—whilst the TLS is meant not to be bound to a specific set of etiologies.

## Limitations and future perspectives

The present study is of course not free of limitations.

In the first place, a number of elements need to be highlighted with regard to the current psychometric analyses.

First, the convergent validity of the TLS has been herewith tested, in HPs, against two measures—*i.e.*, the t-SVF and the TBCLI—that mostly tap into the lexical-semantic component and do not fully cover the wide range of language functions/components covered by the TLS itself. In this respect, it has to be also noted that semantic fluency tasks load on executive functions to a non-negligible extent, albeit to a lesser degree than phonemic fluency ones [72, 73]. Similarly, in neurological patients, convergent validity has been tested against the TICS-L, which, once again, mostly assesses the lexical-semantic component and is thus far from being a comprehensive language index. Hence, even though the TLS proved to be significantly associated with all of the abovementioned measures, there is still a need to further explore its construct validity by employing *ad hoc*, telephone-based language tests that minimally relies on extra-linguistic abilities and that cover the full range of functions/components assessed by the TLS itself.

Second, it has to be noted that the TLS showed acceptable, albeit not high, internal consistency. However, this was hardly unexpected: indeed, its target construct, *i.e.* language, is inherently multi-faceted. Consistently, the adoption of internal consistency as an index of the reliability of cognitive screeners has been questioned – as they often address either multiple cognitive domains/functions or different facets of a given cognitive domain/function that is supposed, but not empirically proven, to be unitary [68].

Third, it is worth noting that the IRT model revealed that the majority of TLS items were overall easy, as well as that only a limited number of them came with a high discriminative power. However, this finding is consistent with the empirical notion according to which language tests are generally not challenging for HPs. At the same time, this should not lead to equate item easiness/low discrimination in HPs to clinical uselessness in patients [74].

Finally, it should be mentioned that the sample from which norms for the advanced CS scoring measures were derived was smaller (*N*=219) when compared to the whole sample of HPs (*N*=480). However, based on the current power analysis, this sample happens to be satisfactory in size, and is larger than that employed for the normative study of the SAND (*N*=134) [33]—which encompasses similar measures. Relatedly, regarding the stratification of the present normative sample, it should be noted that adjustment coefficients might not be validly applicable—and should be thus interpreted cautiously—for those age and education classes which are not herewith represented—*i.e.* individuals with ≤5 years of education and aged up to 60 years for the CS task, and those with ≤5 years of education and aged up to 45 years for the remaining tasks and the TLS-Total.

Some further considerations have to be made with regard to the characteristics of a number of tasks included within the TLS.

First, the restricted time limits that have been set for CS tasks might prevent from collecting meaningful speech samples in patients with severe production deficits (*e.g.* non-fluent aphasias). Thus, although the choice of keeping such a time window as narrow as possible was made in order for the TLS to be less time-consuming as possible, further investigations are needed for determining whether these tasks are actually informative in patients with a severe reduction in speech output.

Second, one might question the adequacy of the Spelling task to the aim of detecting phonological deficits. It is indeed true that, at variance with other languages (such as English), the vast majority of Italian words are featured by predictable phonological-to-orthographic/orthographic-to-phonological conversion rules. Hence, Italian speakers are mostly unfamiliar with oral spelling, this making such task quite challenging for healthy individuals too [75, 76]—as also confirmed by the present IRT analysis showing that Spelling items were among the most difficult ones. In addition, oral spelling engages attentive and executive functions [77]. It follows that, in order to determine whether examinees might present with phonological deficits or not, Spelling scores should not be interpreted alone, but rather in the light of the performance on the other TLS tasks assessing phonology (*i.e.* RoW, RoNW and RoS). Having said that, the Spelling task remains a relevant part of the TLS; a qualitative analysis of the errors on this task can help provide some further insight about the integrity of the phonological structure of single lexical entries.

Third, as is the case for the Token Test from which it takes inspiration, the CML too taps into multiple, extra-linguistic cognitive functions [78]. Indeed, the performance on the Token Test not only depends on the integrity of the morpho-syntactic component, but also on phonological working memory/verbal short-term memory and executive functions [79]. Hence, CML scores need to be interpreted along with the performance on the BDS-T/-WM: in the presence of a defective performance on both the CML and the BDS-T/-WM, examiners should not confidently conclude on the presence of morpho-syntactic deficits. It has to be however noted that the presence of defective BDS-T/-WM performances should lead examiners to interpret with caution the results of all TLS tasks—and not only of the CML—, since working and/or short-term memory impairments negatively affect different language functions.

It should be then borne in mind that the present study is not exhaustive of all the clinimetric and feasibility investigations that are supposed to be performed on a given cognitive screener [68].

First, further studies are mandatory in order to test the diagnostics and cross-sectional feasibility of the TLS in clinical populations whose language impairment, regardless of the aetiology, is confirmed by either a clinical diagnosis or *via* first-/second-level, gold-standard language batteries. such as the SAND [33] or the Aachen Aphasia Test [80], respectively. Indeed, within the present study, only preliminary evidence on the feasibility of the TLS for case-control discrimination and case-finding aims was provided. With this regard, it should be also noted that such results relied on a suboptimal operationalization of the positive state, since the TICS-L is far from being a comprehensive measure of language. Moreover, the present clinical cohort was relatively small in size and highly heterogeneous, and detailed information on both its linguistic and extra-linguistic profile was not collected.

Furthermore, no evidence has been herewith provided on the longitudinal feasibility of the TLS, *e.g.* its responsiveness and susceptibility to practice effects. Future investigations are needed to examine such properties, given that language impairment features itself as a chronic condition in several brain disorders (*i.e.* post-stroke aphasia and primary progressive aphasia) [16]. In this respect, it would also be advisable that reliable change indices are derived and/or that parallel forms are also developed.

## Conclusions

In conclusion, the TLS is a valid, reliable, normed and clinically feasible TBCS test for language deficits. Future studies are nonetheless needed on its clinimetrics and feasibility, both within the cross-sectional and longitudinal dimension, both in HPs and in patients whose core cognitive feature is represented by language impairment.

### Supplementary Information

Below is the link to the electronic supplementary material.

Supplementary file1 (DOCX 50 KB)

Supplementary file2 (XLSX 17 KB)

Supplementary file3 (DOCX 23 KB)

## Data Availability

Datasets associated to the present study have been stored on an open access repository (10.5281/zenodo.10046265) and can be  made available upon reasonable request to the Corresponding Authors.
