# Supplementary material for: The Telephone Language Screener (TLS): standardization of a novel telephone-based screening test for language impairment
Source: Neurol Sci. 2023 Nov 27;45(5):1989–2001. doi: 10.1007/s10072-023-07149-1 (PMC11021315; doi:10.1007/s10072-023-07149-1)
Supplement: Supplementary file 1 — Supplementary file1 (DOCX 50 KB) [file 10072_2023_7149_MOESM1_ESM.docx]

**Development of Telephone Language Screener tasks**

**1. Synopsis**

With the exception of the Connected Speech (CS) task, the development of other tasks begun with an extra and/or non-definitive number/type of items as compared to the final set. This allowed to perform feasibility studies and select the most appropriate items for each task. The feasibility studies for Spelling, Semantic Association (SA), Comprehension and Memory Load (CML), Repetition of Words (RoW), Repetition of Non-Words (RoNW) and Repetition of Sentences (RoS) tasks were conducted on a sample of 29 normotypical individuals (NIs) – 14 males; 15 females; age: 41.1±18.5, *range*=21-85; education: 15.4±3.9, *range*=15-21. The feasibility studies for Naming to Description of Nouns (NtD-N) and Naming to Description of Verbs (NtD-V) tasks were, by contrast, carried out on other two different samples of NIs.

The development Spelling, SA, RoW, RoNW, RoS and NtD-N/-V tasks was pursuant to current psycholinguistic standards, requiring items to be assessed for a number of features known to affect language performances of both normotypical and brain-damaged individuals in a task-specific^[[1]](#footnote-1)^ fashion [Gilardone & Monti, 2019]. Pre-specified thresholds for selected psycholinguistic variables (*e.g.*, frequency) were at times established^[[2]](#footnote-2)^: unless otherwise specified, such an expedient was implemented in order for such tasks to be sensitive not only to moderate-to-severe, but also to mild, language deficits.

The Backward Digit Span (BDS) task has been adapted from Monaco *et al.* (2013) – with the task being thus interrupted after two consecutive errors on a given string level (2 string for each level) –, by however limiting the length of the strings up to 6 digits in order not to overload examinees’ auditory-verbal short-term-memory/phonological working memory.

**2. Connected speech (CS)**

The two items of this task (CSa; CSb) were adapted from Wilson *et al.* (2010), Arcara & Bambini (2016) and Catricalà *et al.* (2017). CSa requires the examinee to describe her/his morning routine and Informative Units (IU) are considered for the scoring. Examinees’ speech samples were audio-recorded in order to allow for their transcriptions and subsequent scoring to be as accurate as possible. After data collection, IUs generated by *N*=401 NIs were qualitatively aggregated into semantic clusters and IU occurring a minimum of 15 times within the normative sample were considered for the scoring (frequency *range*=15-348). Semantic clustering was concurrently performed by two of the Authors, with a third one solving disagreements in classifications. This process resulted in 10 IUs being identified. Whenever examinees reported more than 5 of these IUs, a score of 5 was assigned. CSb did not require any adjustment or feasibility study, as being a mere adaptation to the oral modality of the written description task of the Screening for Aphasia in NeuroDegeneration (SAND) [Catricalà *et al.*, 2017]: IUs for the CSb indeed correspond to those identified by Catricalà *et al.* (2017) – *i.e.*, 3 objects and 3 actions. The scoring corresponds to the number of objects and actions correctly reposted (*range*=0-6; 3 target objects and 3 target actions). The maximum score achievable for the basic CS scoring is thus 11.

The base CS scoring also allows to qualitatively report phonological (*i.e.*, phonemic paraphasias, phonological neologisms, phonological jargon and phonological *conduites d’approche*), lexical-semantic (*i.e.*, circumlocutions, tip-of-the-tongue phenomena, *passe-partout* words, anomic latencies, anomia, semantic paraphasias, verbal paraphasias, semantic jargon and semantic *conduites d’approche*) and morpho-syntactic errors (*i.e.,* agreement errors, inflection errors, omission/misuse of functors, syntactic blendings and use of citation forms) according to the aphasiological literature [Gilardone & Monti, 2019], as well as other qualitative speech features related on the following categories: 1) articulatory difficulties; 2) speech fluency (fluent *vs.* non-fluent)^[[3]](#footnote-3)^; 3) executive-mediated communicative failures (*i.e.*, perseverations, echolalia, coprolalia, recurring utterances and verbal inertia); 4) pragmatic dysfunctions (*i.e.* altered use of referents, rapid topic shifts, tangentiality, hyper-informativity and wrong element order) [Arcara & Bambini, 2016; Catricalà *et al.*, 2017; Gilardone & Monti, 2019].

The advanced CS scoring then allows for 1) an in-depth, qualitative report of dysarthric features (*i.e.*, slurred speech, dysphonia, hypernasality and pace alterations) and apraxia of speech (*i.e.*, scanning speech, dysprosody, filled pauses as well as alterations and variability in the production of the phonemes) according to the current literature on motor speech disorders [Strand *et al.*, 2014; Gilardone & Monti, 2019] and 2) the derivation of a number of quantitative measures for speech analysis, including both unitary (*i.e.*, the total number of words and sentences) and “ratio” measures (*i.e.*, the number of nouns, verbs and function words out of the total number of words) [Wilson *et al.*, 2010; Catricalà *et al.*, 2017; Gilardone & Monti, 2019]. These last quantitative measures were selected due to the fact that they are acknowledge to be useful to the aim of assessing lexical-semantic and morpho-syntactic components in connected speech samples [Wilson *et al.*, 2010; Catricalà *et al.*, 2017; Gilardone & Monti, 2019].

**3. Spelling**

Stimuli were either derived from Luzzatti *et al.*’s (1994) writing-to-dictation task or generated *ex novo* to fit the following classes: 1) regular words with no one-to-one phonological-orthographic correspondence and 2) irregular words.

A preliminary set of 15 words were administered to the feasibility sample to compute response agreement rates. A set of 10 words was identified yielding a response agreement ≥83%. In order for stimuli to be relatively sensitive, the following psycholinguistic variables were addressed: word frequency ≤688 (*range*=30-688; as computed through the SUBTLEX-IT corpus) [Crepaldi *et al.*, 2016]; at least one consonant cluster (including doubles); length in letters and syllables ≥5 (*range*=5-7) and 2, respectively. Word frequency was considered since high frequency words are processed faster than low-frequency words [Brysbaert *et al*., 2017], while word length and phonological complexity due to the fact that these features place a load on phonological working memory processes [Gilardone & Monti, 2019]. The abovementioned thresholds and ranges for word frequency and word length were set in order for the task not to easily reach either ceiling or floor effects. This was also the rationale underlying the choice for the selection of words with medium phonological complexity – *i.e.*, including at least one consonant cluster and not merely having a consonant-vocal-consonant-vocal structure [Luzzatti *et al.*, 1994].

**4. Semantic Association**

This task was adapted from Luzzatti *et al.* (2020) and the relevant procedures were carried out in accordance with the principles outlined by Catricalà *et al.* (2013). A set of 12 target-probe-distractor triplets was initially selected, considering the following psycholinguistic variables: disyllabic words with low phonological complexity – in order for this task not to place a heavy burden on verbal short-term memory/phonological working memory functions – , high level of concreteness and imageability – in order for verbally mediated associations to be performable similarly to those based on visual stimuli [Luzzatti *et al.*, 2020] – and homogeneity in terms of age of acquisition^[[4]](#footnote-4)^ – as words acquired earlier in life are faster to be processed than those acquired later [Brysbaert *et al*., 2000] – within each item [Dell’Acqua *et al.*, 2000; Della Rosa *et al.*, 2010; Navarrete *et al.*, 2019]. In addition, the target was selected in order for it to have always a shorter semantic distance with the probe when compared to the distractor [Marelli, 2017]. According to Luzzatti *et al.* (2020), triplets were of three types, based on the nature of the semantic association: categorical, functional, and encyclopedic/visual-encyclopedic. The last three categories were aggregated for the sake of parsimony – in the light of the similarity between the encyclopedic and visual-encyclopedic categories. Differences semantic association types have been considered due to the fact that they can be selectively damaged based on the involved neural networks [Luzzatti *et al.*, 2020].

After the feasibility study, six items were selected, two for each category – *i.e.,* categorical *vs.* functional/encyclopedic/visual-encyclopedic. In particular, items without ceiling effects in agreement rates were chosen (*range*=93%-96%), with the exception of one item (100%). With respect to the selected 6 items, semantic distance from the target was significantly higher (*t*(10)=3.83; *p*=.003) for the distractor (*M*=.87; *SE=*.02) when compared to the probe (*M*=.7; *SE*=.04). Age of acquisition was homogeneous across the 6 items (*F*(5,12)=2.77; *p*=.068).

**5. Naming to Description of Nouns**

The feasibility study for the NtD-N were pursuant to Catricalà *et al.*’s (2013) procedures. A set of twenty preliminary items was selected considering the following psycholinguistic properties: low frequency (*i.e.*, ≤2.5) – as high frequency words are processed faster than low-frequency words [Brysbaert *et al*., 2017], and thus in order for the task not to easily reach ceiling effects –, number of features related to the word to name ≥16 (which was deemed as a sufficient number of characteristics related to the word to name so that the vast majority of examinees could agree upon the identification of the word itself), concreteness, abstractness and imageability – as a better performance is typically related to words with higher imageability and/or higher concreteness [Nickels & Howard, 1995; Kiran *et al.*, 2009] –, familiarity – as highly familiar words are retrieved faster than less familiar words [Lewellen *et al*., 1993] –, age of acquisition (in weeks) – as words acquired earlier in life are more easily named when compared to those acquired later [e.g., Morrison & Ellis, 2000] –, context availability – since, in accordance with Schwanenflugel *et al*. (1988), concrete words activate a broader contextual verbal support resulting in faster processing –, acquisition modality – since the way through which a given word is learned (*e.g.*, from first-person experiences in the real world *vs.* from school) may influence individuals’ ability to name it [Della Rosa *et al.*, 2003] –, number of syllables and word length – as longer words are more difficult to be retrieved than shorter ones [Klapp *et al*., 1973] –, typicality – which has been shown to influence performance in language tasks with faster responses for high than low typicality items [Rossiter, & Best, 2013; Kiran *et al.*, 2007] –, and lastly mean time of naming (in seconds)^[[5]](#footnote-5)^ – as it may influence naming performance [Ferrand et *et al.*, 2011] - [Dall’Acqua *et al.*, 2000; Della Rosa *et al.*, 2003]. All of the values for the abovementioned variables are taken from Dall’Acqua *et al.*, 2000 and Della Rosa *et al.*, 2003, with the exception of the number of features – which was taken from Catricalà *et al*. (2013; 2015) – and semantic relevance – which was re-calculated solely based on the parameters of the features that were actually selected for each item. In addition, the items were half living and half non-living, and, at independent-sample *t-*tests, psycholinguistic properties of the two groups were not significantly different (*p*s≥.119).

The feasibility study was carried out in a sample of 27 NIs (16 females, 11 males; age: 32.4±13, *range*=18-63; education: 16.5±2.9, *range*=10-21).

After the feasibility study, six items were selected, three for each category, considering the highest level of agreement among participants (≥88%). Psycholinguistic features of such items are reported in Table 1, separately for living and non-living ones. The presentation order of each item was organized as follows:  nouns of the same category or with the same initial were not consecutive and semantic distance between contiguous nouns was greater than .8.

**Table 1**. Psycholinguistic features of the final set of items included in the Naming to Description of Nouns task of the Telephone Language Screener.

| **Psycholinguistic feature** | **Living nouns (*N*=3)** | **Non-living nouns (*N*=3)** |
| --- | --- | --- |
| Semantic relevance | 27.5±4 (22-32.2) | 30±3.3 (26.2-32.4) |
| Number of features | 27.8±4.4 (23-32) | 25±2 (23-27) |
| Concreteness | 686.5±13.1 (668.8-700) | 687.4±4.3 (684.6-692.3) |
| Imageability | 678.4±16.1 (661.8-694.7) | 683.4±10.1 (672-691.3) |
| Familiarity | 571.5±74.3 (481.8- 645.5) | 513.2±43.8 (481.8-563.2) |
| Age of acquisition (weeks) | 198.1±8.1 (191.7-210) | 230±33.6 (208.8-268.8) |
| Context availability | 641.5±27.3 (617.6-673.5) | 648.4±18.6 (632.4-668.8) |
| Abstractness | 100.7±1.5 (100-102.9) | 104.6±1.5 (102.9-105.9) |
| Modality of acquisition | 227.2±63.1 (144-296) | 198±20.9 (175-215.8) |
| Length in letters | 8.5±1 (8-10) | 7.3 ± .6 (7-8) |
| Length in syllables | 3.8±.5 (3-4) | 3±0 |
| Word frequency | 1.8±.3 (1.4-2.2) | 1.3±.1 (1.2-1.4) |
| Typicality | 4.6±1.3 (2.7- 5.5) | 4.5±1.6 (3.1-6.2) |
| Naming reaction times (seconds) | 1013.8±262.2 (655-1280) | 754.3±91.5 (651-825) |

**Notes.** Non-aggregated values (not shown) are taken from Dall’Acqua *et al.* (2000) and Della Rosa *et al.* (2003), except for the number of features – which was taken from Catricalà *et al.* (2013; 2015) – and semantic relevance – which was calculated solely based on the parameter that were actually selected for each item *via* the following formula: *dominance**(log_2_( *distinctiveness*). Continuous variables are shown as *M*±*SD* (*range*).

**6. Naming to Description of Verbs**

The feasibility study for the NtD-V were pursuant to Catricalà *et al.*’s (2013) procedures. A set of 20 verbs with low frequency (*i.e.*, ≤15)^[[6]](#footnote-6)^ was selected from Crepaldi *et al.* (2006), considering the following psycholinguistic properties: length in letters and syllables, age of acquisition (in years), naming agreement, imageability, typicality, familiarity^[[7]](#footnote-7)^, argument structure type (transitive *vs.* intransitive) and actionality [Crepaldi *et al.*, 2006; Aiello *et al.*, 2022]. These last two features were considered due to the fact that 1) different argument structure types are acknowledged to place different processing loads (according to the Argument Structure Complexity Hypothesis [Thompson, 2003; den Ouden *et al.*, 2009]; *e.g.*, transitive verbs with three mandatory arguments – such as “to send” within the sentence “*she* sent *me* a *postcard*” – place a higher processing load than intransitive verbs with a single mandatory argument – such as “to sleep” within the sentence “*he* is sleeping”) and 2) the degree of motor content within a given verb (*i.e.*, their actionality) might influence individuals’ ability to retrieve them (*e.g.*, low-actionality verbs are easier to be processed as they are more concrete and imageable) [Aggujaro *et al.*, 2006; Aiello *et al.*, 2022a; 2023]. Psycholinguistic properties for such items were taken from Crepaldi *et al.* (2006), Aiello (2022) and Aiello *et al.* (2022a). All the properties were not significantly different between transitive and intransitive verbs at independent-sample *t-*tests (*p*s≥.105).

Subsequently, 25 NIs (14 females, 11 males; mean age: 30, *range*=16-62; mean education: 16.4, *range*=10-20) were asked to list as many features as possible in order to describe the set of 20 verbs. To facilitate the feature production, a series of cues was provided to participants (*e.g.,* “*When, where, who, associated objects, goal*?”). Data were analyzed following the criteria for the features categorization of McRae *et al.* (1997). Features generated by fewer than two NIs were excluded. All the similar features were aggregated and classified into one of seven possible category types: 1) subject, 2) object, 3) physical and 4) temporal context, 5) cause, 6) consequence and 7) attribute. For each feature, the following properties were calculated: 1) dominance, *i.e.* the number of NIs who listed a specific feature for a specific concept (*range*=2-24, *M*=4.67) [Garrard, *et al.*, 2001; McRae *et al.*, 2005; Ashcraft, 1978]; 2) frequency (*range*=1-5, *M*=1.26), *i.e.* the number of concepts for which a given feature appeared (with respect to each category and to all concepts from the database); 3) two different measures of distinctiveness (*range*=4-20, *M*=18.4): the number of concepts for which the semantic feature appears, divided by the total number of concepts in the database [Devlin *et al.*, 1998; McRae *et al.,* 2005], and ‘‘the proportion of concepts within a category for which the feature in question was generated’’ [Garrard *et al.*, 2001]; 4) semantic relevance (*range*=4-104, *M*=19.1), as a non-linear combinations between dominance and distinctiveness [Sartori *et al.*, 2004; Mechelli *et al.*, 2006].

After feature selection, a definition for each verb was created following this structure: “*action that*”, followed by 4 features different for category types.

The twenty preliminary items were administered to a different sample of 23 NIs (13 females, 10 males; mean age: 30, *range*=18-60; mean education: 15, *range*=10-21) to choose the final items.  After the preliminary check, six items were selected, three transitive and three intransitive verbs, considering the highest level of agreement among NIs (≥96%). Psycholinguistic features of such items are reported in Table 2, separately for transitive and intransitive ones. The order of single items was organized as follows: verbs of the same category or with the same initial were administered not consecutively, and semantic distance values between contiguous items had to be greater than .8.

**Table 2**. Psycholinguistic features of the final set of items included in the Naming to Description

of Verbs task of the Telephone Language Screener.

| **Psycholinguistic feature** | **Intransitive verbs (*N*=3)** | **Transitive verbs (*N*=3)** |
| --- | --- | --- |
| Word frequency | 3.7±4.6 (1- 9) | 0 |
| Length in letters | 9.3±1.5 (8-11) | 9±2.6 (6-11) |
| Length in syllables | 3.7±.6 (3-4) | 4±1 (3-5) |
| Age of acquisition (years) | 3.9±1.8 (2.4-5.8) | 3.9±1.2 (2.6-5) |
| Naming agreement (%) | 100%±.1% (90%-100%) | 100% |
| Imageability | 5±1 (3.9-5.7) | 4.8±.8. (4-5.6) |
| Typicality | 5.7±.65 (5.2-6.4) | 6.1±.8 (5.2-6.7) |
| Familiarity | 5±1.3 (4-6.5) | 4.7±1 (4-5.9) |
| Actionality | 4.2±.6 (3.5-4.6) | 4.9±.3 (4.5- 5.1) |
| Number of features* | 18±5.3 (12-22) | 18.7±2.1 (17-21) |
| Feature dominance* | 6.8±1.7 (5.5-8.7) | 6.9±.9 (6.1-7.8) |
| Feature frequency* | 1.2±.1 (1-1.2) | 1.2±.2 (1-1.4) |
| Feature distinctiveness* | 19.3±.6 (19-20) | 18.7±1.2. (18-20) |
| Semantic relevance of features* | 40.5±10.6 (28.5-48.6) | 49±3.5 (46.5-53) |

**Notes.** *values empirically derived within the feasibility study. Non-aggregated values (not shown) are taken from Crepaldi *et al.* (2006), Aiello (2022) and Aiello *et al.* (2022). Continuous variables are shown as *M*±*SD* (*range*).

**7. Comprehension and Memory Load**

A set of 6 items was anew-developed and selected for this task, divided into pairs of two by difficulty and number of requests – inspired to the Token Test [De Renzi *&* Faglioni, 1978]. Items 1a and 1b have simple biphasic orders with coordination bond – *e.g.,* “*tap once on the table and tell what color it is the snow*”; Items 2a and 2b, on the other hand, are two-phase orders with a subordination link (*e.g.*: “*if the dog is an animal, say the day that comes before Friday*”). Finally, Items 3a and 3b correspond to three-phase orders which embed a requirement referred to the previous one – *e.g*., “*say what color the banana is after saying the letter "A", rather, the letter "O"*”*.*

The score for each sentence is assigned based on the understanding of each underlying phase/section. During the feasibility study on the NI sample, the task only underwent a main change related to the replacement of the response modality for a number of items. Indeed, originally, some items required telephone-tapping [Brandt *et al*, 1988; Christodoulou *et al.*, 2016]: however, such a requirement was eliminated from the final item set and replaced with a "*tap on the table*" order [Aiello *et al.*, 2022b], due to the fact that it was deemed much more difficult for the examiner’s standpoint to hear the tapping on the telephone. It was deemed that such difficulties were linked to discrepancies among receivers’ telephones – *e.g.*, type of screen and telephone, position (near or far from the microphone).

**8. Repetition of words**

The items from this task were derived from the SAND [Catricalà *et al.*, 2017]. A preliminary set of 12 words with low frequency (*i.e.*, ≤5) and low phonological neighborhood density (*i.e.* ≤6) [De Mauro *et al*., 1993] was selected, and the following psycholinguistic variables were retrieved: concreteness (concrete or abstract) – as abstract words are more difficult to be processed and thus repeated than concrete ones [Hoffman, 2016] – and word length (short or long) – as shorter words are easier processed and repeated than the longer ones – [Catricalà *et al.*, 2017]. The consideration of such variables allowed for the task not to be to prone to be subjected to either ceiling or floor effects.

The preliminary set was administered to the feasibility sample. Then, the items with the highest agreement (≥90%; *range*=92%-100%) among NIs were selected. The final version consists of six items (4/6 concrete and 2/6 abstract words, 4/6 long and 2/6 short) all derived from the SAND [Catricalà *et al*., 2017] with the exception of one word (*i.e.,* “*frode*”) which was substituted with another (*i.e.,* “*crepa*”) due to the difficulty of comprehension by examinees’ standpoint *via* telephone detected during the feasibility study.

**9. Repetition of Non-words**

The items from this task were derived from the SAND [Catricalà *et al.*, 2017]. A set of 12 preliminary items was selected considering two psycholinguistic variables: word length (as accuracy decreases as non-word length increases [Coady & Evans, 2008]) and phonological closeness to words (close or distant, with non-words being phonologically more similar, and thus close, to words being more difficult to be repeated correctly [Gathercole *et al*., 1991]) [Catricalà *et al.*, 2017]. The consideration of such variables allowed for the task not to be to prone to be subjected to either ceiling or floor effects.

The preliminary set was administered to the feasibility sample and the items with the highest agreement (≥90%; *range*=93%-97%) among NIs were selected. The final version consists of five items, all derived from the SAND [Catricalà *et al.*¸2017] – *i.e.*, 2 long non-words close to a word, one long non-word distant from a word and 2 short non-words and close to a word).

**10. Repetition of sentences**

The items from this task were derived from the SAND [Catricalà *et al.*, 2017]. A set of 6 preliminary sentences was selected considering three psycholinguistic variables: sentence length (short or long, since longer sentences place a heavier load on working memory than shorter ones), semantic predictability (as sentence processing is easier in more predictable sentences [Roland *et al*., 2011]) and grammatical complexity (since more complex sentences may be more difficult to be correctly repeated). The consideration of such variables allowed for the task not to be to prone to be subjected to either ceiling or floor effects.

The preliminary set was administered the feasibility sample and the sentences with the highest agreement (≥86%; *range*=86%-100%) among NIs were selected. The final version consists of three sentences: all unpredictable – to increase task complexity –, 2 long and one short.

**References**

1. Aiello, E. N. (2022). *Action-Verb Naming Test - Psycholinguistic variables.* Retrieved October 25, 2022 from <https://osf.io/9ukxj/>
2. Aiello, E. N., Grosso, M., Di Liberto, A., Andriulo, A., Buscone, S., Caracciolo, C., Ottobrini, M., Luzzatti, C. (2022a). Disembodying language: Actionality does not account for verb processing deficits in Parkinson's disease. *Journal of Neurolinguistics*, *61*, 101040.
3. Aiello, E.N., Pucci, V., Diana, L., Niang, A., Preti, A. N., Delli Ponti, A., Sangalli, G., Scarano, S., Tesio, L., Zago, S., Difonzo, T., Appollonio, I., Mondini, S., Bolognini, N. (2022b). Telephone-based Frontal Assessment Battery (t-FAB): standardization for the Italian population and clinical usability in neurological diseases*.* *Aging and Clinical Experimental Research*, *34*, 1635-1644.
4. Aiello, E. N., Pain, D., Gallucci, M., Feroldi, S., Guidotti, L., Mora, G., & Luzzatti, C. (2023). Rethinking motor region role in verb processing: Insights from a neurolinguistic study of noun-verb dissociation in amyotrophic lateral sclerosis. *Journal of Neurolinguistics*, *66*, 101124.
5. Aggujaro, S., Crepaldi, D., Pistarini, C., Taricco, M., Luzzatti, C. (2006). Neuro-anatomical correlates of impaired retrieval of verbs and nouns: Interaction of grammatical class, imageability and actionality. *Journal of Neurolinguistics*, *19*, 175-194.
6. Arcara, G., Bambini, V. (2016). A Test for the Assessment of Pragmatic Abilities and Cognitive Substrates (APACS): Normative Data and Psychometric Properties. *Frontiers in Psychology, 7*, 70.
7. Ashcraft, M. H. (1978). Property norms for typical and atypical items from 17 categories: A description and discussion. *Memory & Cognition*, *6*, 227-232.
8. Brandt, J., Spencer, M., Folstein, M. (1988). The telephone interview for cognitive status. *Neuropsychiatry, Neuropsychology and Behavioural Neurology*, *1*, 111-117.
9. Brysbaert, M., Van Wijnendaele, I., De Deyne, S. (2000). Age-of-acquisition effects in semantic processing tasks. *Acta psychologica, 104,* 215–226.
10. Brysbaert, M., Mandera, P., Keuleers, E. (2018). The Word Frequency Effect in Word Processing: An Updated Review. *Current Directions in Psychological Science*, *27*, 45-50.
11. Catricala, E., Della Rosa, P. A., Ginex, V., Mussetti, Z., Plebani, V., & Cappa, S. F. (2013). An Italian battery for the assessment of semantic memory disorders. *Neurological Sciences*, *34*, 985-993.
12. Catricalà, E., Ginex, V., Dominici, C., Cappa, S. F. (2015). A new comprehensive set of concept feature norms. *Revista Portuguesa de Psicologia*, *44*, 111-120.
13. Catricalà, E., Gobbi, E., Battista, P., Miozzo, A., Polito, C., Boschi, V., ... & Garrard, P. (2017). SAND: a Screening for Aphasia in NeuroDegeneration. Development and normative data. *Neurological Sciences*, *38*, 1469-1483.
14. Christodoulou, G., Gennings, C., Hupf, J., Factor-Litvak, P., Murphy, J., Goetz, R. R., Mitsumoto, H. (2016). Telephone based cognitive-behavioral screening for frontotemporal changes in patients with amyotrophic lateral sclerosis (ALS). *Amyotrophic Lateral Sclerosis and Frontotemporal Degeneration*, *17*, 482-488.
15. Clough, S., Gordon, J. K. (2020). Fluent or nonfluent? Part A. Underlying contributors to categorical classifications of fluency in aphasia. *Aphasiology*, *34*, 515-539.
16. Coady, J. A., Evans, J. L. (2008). Uses and interpretations of non-word repetition tasks in children with and without specific language impairments (SLI). *International journal of language & communication disorders*, *43*(*1*), 1–40.
17. Crepaldi, D., Aggujaro, S., Arduino, L. S., Zonca, G., Ghirardi, G., Inzaghi, M. G., ... & Luzzatti, C. (2006). Noun–verb dissociation in aphasia: The role of imageability and functional locus of the lesion. *Neuropsychologia*, *44*, 73-89.
18. Crepaldi, D., Amenta, S., Mandera, P., Keuleers, E., and Brysbaert, M. SUBTLEX-IT (2015, September 10-12). *Subtitle-based word frequency estimates for Italian.* Annual Meeting of the Italian Association for Experimental Psychology, Rovereto.
19. De Renzi, E., Faglioni, P. (1978). Normative data and screening power of a shortened version of the Token Test. *Cortex*, *14*, 41-49.
20. Della Rosa, P. A., Catricalà, E., Vigliocco, G., Cappa, S. F. (2010). Beyond the abstract-concrete dichotomy: mode of acquisition, concreteness, imageability, familiarity, age of acquisition, context availability, and abstractness norms for a set of 417 Italian words. *Behavior Research Methods*, 42, 1042–1048.
21. Dell'Acqua, R., Lotto, L., Job, R. (2000). Naming times and standardized norms for the Italian PD/DPSS set of 266 pictures: direct comparisons with American, English, French, and Spanish published databases. Behavior Research Methods, Instruments, & Computers, *32*, 588–615.
22. den Ouden, D. B., Fix, S., Parrish, T. B., Thompson, C. K. (2009). Argument structure effects in action verb naming in static and dynamic conditions. *Journal of Neurolinguistics*, *22*, 196-215.
23. Devlin, J. T., Gonnerman, L. M., Andersen, E. S., Seidenberg, M. S. (1998). Category-specific semantic deficits in focal and widespread brain damage: A computational account. *Journal of cognitive Neuroscience*, *10*, 77-94.
24. Ferrand, L., Brysbaert, M., Keuleers, E., New, B., Bonin, P., Méot, A., Augustinova, M., Pallier, C. (2011) Comparing word processing times in naming, lexical decision, and progressive demasking: evidence from Chronolex. *Frontiers in Psychology*, *2*, 306.
25. Garrard, P., Lambon Ralph, M. A., Hodges, J. R., Patterson, K. (2001). Prototypicality, distinctiveness, and intercorrelation: Analyses of the semantic attributes of living and nonliving concepts. *Cognitive Neuropsychology*, *18*, 125-174.
26. Gordon, J. K., Clough, S. (2020). How fluent? Part B. Underlying contributors to continuous measures of fluency in aphasia. *Aphasiology*, *34*, 643-663.
27. Gordon, J. K., Clough, S. (2022). How Do Clinicians Judge Fluency in Aphasia?. *Journal of Speech, Language, and Hearing Research*, *65*, 1521-1542.
28. Gathercole S. E., Willis C., Emslie H., Baddeley A. D. (1991). The influences of number of syllables and word-likeness on children's repetition of nonwords. *Applied Psycholinguistics*,*12*, 349–367.
29. Kiran S., Ntourou K., Eubanks M. (2007). Effects of typicality on category verification in inanimate categories in aphasia. *Aphasiology*, *21*, 844–867.
30. Kiran, S., Sandberg, C., Abbott, K. (2009). Treatment for lexical retrieval using abstract and concrete words in persons with aphasia: Effect of complexity. *Aphasiology, 23*, 835–853.
31. Klapp, S. T., Anderson, W. G., Berrian, R. W. (1973). Implicit speech in reading, reconsidered. *Journal of Experimental Psychology*, *100*, 368–374.
32. Hoffman P. (2016). The meaning of 'life' and other abstract words: Insights from neuropsychology. *Journal of neuropsychology*, *10*, 317–343.
33. Luzzatti, C., Laiacona, M., Allamano, N., De Tanti, A., Inzaghi, M. G., Lorenzi, L. (1994). An Italian test for the diagnosis of acquired writing disorders: construction principles and normative data. *Ricerche di Psicologia*, *18*, 137-160.
34. Luzzatti, C., Mauri, I., Castiglioni, S., Zuffi, M., Spartà, C., Somalvico, F., Franceschi, M. (2020). Evaluating semantic knowledge through a semantic association task in individuals with dementia. *American Journal of Alzheimer's Disease & Other Dementias®*, *35*, 1533317520917294.
35. Marelli, M. (2017). Word-Embeddings Italian Semantic Spaces: A semantic model for psycholinguistic research. *Psihologija, 50*, 503–520.
36. McRae, K., De Sa, V. R., & Seidenberg, M. S. (1997). On the nature and scope of featural representations of word meaning. *Journal of Experimental Psychology: General*, *126*, 99-130.
37. McRae, K., Cree, G. S., Seidenberg, M. S., McNorgan, C. (2005). Semantic feature production norms for a large set of living and nonliving things. *Behavior Research Methods*, *37*, 547-559.
38. Mechelli, A., Sartori, G., Orlandi, P., Price, C. J. (2006). Semantic relevance explains category effects in medial fusiform gyri. *Neuroimage*, *30*, 992-1002.
39. Monaco, M., Costa, A., Caltagirone, C., Carlesimo, G. A. (2013). Forward and backward span for verbal and visuo-spatial data: standardization and normative data from an Italian adult population. *Neurological Sciences*, *34*, 749-754.
40. Morrison, C. M., Ellis, A. W. (2000). Real age of acquisition effects in word naming and lexical decision. *British Journal of Psychology*, *91*, 167–180.
41. Navarrete, E., Arcara, G., Mondini, S., Penolazzi, B. (2019). Italian norms and naming latencies for 357 high quality color images. *PLoS One, 14*, e0209524.
42. Nickels, L., Howard, D. (1995). Aphasic naming: what matters?. *Neuropsychologia*, *33*, 1281–1303.
43. Roland, D., Yun, H., Koenig, J. P., Mauner, G. (2012). Semantic similarity, predictability, and models of sentence processing. *Cognition*, *122*, 267-279.
44. Rossiter, C., Best, W. (2013). "Penguins don't fly": An investigation into the effect of typicality on picture naming in people with aphasia. *Aphasiology*, *27*, 784–798.
45. Sartori, G., Lombardi, L. (2004). Semantic relevance and semantic disorders. *Journal of Cognitive Neuroscience*, *16*, 439-452.
46. Schwanenflugel, P. J., Harnishfeger, K. K., Stowe, R. W. (1988). Context availability and lexical decisions for abstract and concrete words. Journal of Memory & Language, 27, 499-520.
47. Strand, E. A., Duffy, J. R., Clark, H. M., Josephs, K. (2014). The Apraxia of Speech Rating Scale: A tool for diagnosis and description of apraxia of speech. *Journal of Communication Disorders*, *51*, 43-50.
48. Thompson, C. K. (2003). Unaccusative verb production in agrammatic aphasia: The argument structure complexity hypothesis. *Journal of Neurolinguistics*, *16*, 151-167.
49. Wilson, S. M., Henry, M. L., Besbris, M., Ogar, J. M., Dronkers, N. F., Jarrold, W., ... & Gorno-Tempini, M. L. (2010). Connected speech production in three variants of primary progressive aphasia. *Brain*, *133*, 2069-2088.

1. It would be beyond the scopes of this document to provide the Reader with a thorough explanation of the rationale underlying the selection of a specific set of psycholinguistic variables for the evaluation of each task at hand. To this last aim, the Reader is however referred to the following papers: 1) Spelling: Luzzatti *et al.* (1994); 2) Semantic Association: Luzzatti *et al.* (2020) and Catricalà *et al.* (2013; 2017); 3) RoW, RoNW and RoS: Catricalà *et al.* (2017); 4) NtD-N/-V: Crepaldi *et al.* (2006); Aiello *et al.* (2022a); Catricalà *et al.* (2013). [↑](#footnote-ref-1)
2. Such thresholds were identified *ad hoc* based on the specific set(s) of psycholinguistic variables that was/were addressed when developing a given task. [↑](#footnote-ref-2)
3. In this respect, it has to be noted that a dichotomous classification of speech fluency is not always straightforward, given that this construct relies on multiple features of examinees’ speech output related to articulatory processes, phonological encoding, lexical retrieval abilities and morpho-syntactic skills [Gordon & Clough, 2022]. Hence, the classification of speech output as either “fluent” or “non-fluent” should underlie an in-depth, multi-faceted observational analysis – whose description would be, however, beyond the scope of this work, since such a measure is not enclosed within the quantitative scoring of the TLS. Examiners might refer to the recent works by Gordon & Clough (2020a; 2020b; 2022) for a proper appraisal of this matter. [↑](#footnote-ref-3)
4. Age of acquisition values for those stimuli addressed within the development of the Semantic Association task were not available within a single published work; hence, the present Authors referred to three different sets – namely, those by Dell’Acqua *et al.* (2000), by Della Rosa *et al.* (2010) and by Navarrete *et al.* (2019). However, since this psycholinguistic variable has been scaled differently in Dell’Acqua *et al.*’s (2000) and Navarrete *et al.*’s (2019) works (*i.e.*, 9-point scale) when compared to that by Della Rosa *et al.* (2010) (*i.e.*, 7-point scale), a proportion has been applied, for homogeneity purposes, in order for all of these values to be expressed on a 7-point scale. [↑](#footnote-ref-4)
5. It has to be noted that typicality and mean naming time measures reported in Dell'Acqua *et al*. (2000) are related to the picture depicting the word and not to the written word. [↑](#footnote-ref-5)
6. The rationale underlying the selection of low-frequency words was the same as for the NtD-N task. [↑](#footnote-ref-6)
7. These psycholinguistic properties were considered for the same underlying reasons as for the Naming to Description of Noun task, as affecting not only noun-naming but also verb-naming abilities [ [↑](#footnote-ref-7)
