# Supplementary material for: The Telephone Language Screener (TLS): standardization of a novel telephone-based screening test for language impairment
Source: Neurol Sci. 2023 Nov 27;45(5):1989–2001. doi: 10.1007/s10072-023-07149-1 (PMC11021315; doi:10.1007/s10072-023-07149-1)
Supplement: Supplementary file 3 — Supplementary file3 (DOCX 23 KB) [file 10072_2023_7149_MOESM3_ESM.docx]

**Supplementary Table 1.** Sample stratification for age, education, and sex.

| Male/female | Age | | | | | | |
| --- | --- | --- | --- | --- | --- | --- | --- |
| Education | **30≤** | **31-45** | **46-60** | **61-70** | **71-80** | **≥81** | **Total** |
| 5≤ | 0/0 | 0/0 | 0/1 | 0/4 | 0/9 | 3/11 | 3/25 |
| 6-8 | 4/1 | 2/3 | 14/22 | 6/8 | 6/7 | 0/6 | 32/47 |
| 9-12 | 2/0 | 2/2 | 5/18 | 6/6 | 4/6 | 0/1 | 19/33 |
| 13-16 | 34/39 | 9/12 | 34/52 | 10/16 | 7/6 | 3/1 | 97/126 |
| ≥17 | 13/19 | 5/4 | 11/19 | 7/12 | 3/1 | 1/3 | 40/58 |
| Total | 53/59 | 18/21 | 64/112 | 29/46 | 20/29 | 7/22 | 191/289 |

**Supplementary Table 2.** TLS tasks and corresponding IPCLI measures.

| TLS (*range*) | IPCLI (*range*) |
| --- | --- |
| Spelling (0-10) | Spelling - ECAS (0-12) |
| Semantic Association (0-6) | Semantic Association - SAND (0-4) |
| Naming to Description of Nouns (0-6) | Noun-Naming - ENPA (0-10) |
| Naming to Description of Verbs (0-6) | Verb-Naming - ENPA (0-10) |
| Repetition of Words (0-6) | Repetition of Words - SAND (0-6) |
| Repetition of Non-Words (0-5) | Repetition of Non-Words - SAND (0-4) |
| Repetition of Sentences (0-3) | Repetition of Sentences - SAND (0-6) |
| Comprehension and Memory Load (0-15) | Comprehension of Sentences - SAND (0-8) |
| Informative Units (0-11) | Informative Units - SAND (0-8) |
| Notes. TLS=Telephone Language Screener; IPCLI=In-Person Composite Language Index; ECAS=Edinburgh Cognitive and Behavioural ALS Screen; SAND=Screening for Aphasia in NeuroDegeneration; ENPA=Esame NeuroPsicologico per l’Afasia. | |

**Supplementary Table 3.** Demographic and language measures of the HP subsample undergone the IPCLI.

| *N* | 79 |
| --- | --- |
| Age (years) | 50.8±18 (19-84) |
| Sex (male/female) | 32/47 |
| Education (years) | 12.8±3.5 (5-22) |
| TLS |  |
| Total | 61.7 ± 4.6 (42-68) |
| Spelling | 9±1.3 (4-10) |
| Semantic Association | 5.9±.3 (4-6) |
| Naming to Description of Nouns | 5.7±.6 (3-6) |
| Naming to Description of Verbs | 5.5±.9 (1-6) |
| Repetition of Words | 5.8±.5 (4-6) |
| Repetition of Non-Words | 4.1±1 (0- 5) |
| Repetition of Sentences | 2.7±.5 (1-3) |
| Comprehension and Memory Load | 14.3±1.6 (6-15) |
| Informative Units | 8.7±2.2 (0-11) |
| IPCLI |  |
| Total | 63.7±3.7 (49-68) |
| Spelling - ECAS | 10.9±1.7 (5-12) |
| Semantic Association - SAND | 3.8±.5 (2-4) |
| Noun-Naming - ENPA | 9.9 ± .2 (9-10) |
| Verb-Naming - ENPA | 9±1 (5-10) |
| Repetition of Words - SAND | 6±.1 (5-6) |
| Repetition of Non-Words - SAND | 3.5±.7 (1-4) |
| Repetition of Sentences - SAND | 5.6±.7 (3-6) |
| Comprehension of Sentences - SAND | 7.9±.4 (6-8) |
| Informative Units - SAND | 7.1±1.5 (0-8) |
| Notes. TLS=Telephone Language Screener; HP=healthy participant; IPCLI=In-Person Composite Language Index; ECAS=Edinburgh Cognitive and Behavioural ALS Screen; SAND=Screening for Aphasia in NeuroDegeneration; ENPA=Esame NeuroPsicologico per l’Afasia. | |

**Supplementary Table 4.** Stratification for age, education, and sex of the HP subsample (*N*=219) from which norms for the advanced CS scoring measures were derived.

| Male/female | Age | | | | | | |
| --- | --- | --- | --- | --- | --- | --- | --- |
| Education | **30≤** | **31-45** | **46-60** | **61-70** | **71-80** | **≥81** | **Total** |
| 5≤ | 0/0 | 0/0 | 0/0 | 0/2 | 0/2 | 0/5 | 0/9 |
| 6-8 | 4/0 | 1/2 | 7/12 | 3/1 | 1/2 | 0/3 | 16/20 |
| 9-12 | 0/0 | 0/1 | 2/11 | 4/5 | 2/0 | 0/0 | 8/17 |
| 13-16 | 10/13 | 8/10 | 16/26 | 3/13 | 1/3 | 0/1 | 38/66 |
| ≥17 | 4/6 | 4/3 | 6/5 | 6/7 | 1/0 | 1/2 | 22/23 |
| Total | 18/19 | 13/16 | 31/54 | 16/28 | 5/7 | 1/11 | 84/135 |

**Notes.** CS=Connected Speech; HP=healthy participant.

**Supplementary Table 5.** Demographic and advanced CS scoring measures of the HP subsample from which their norms were derived.

| *N* | 219 |
| --- | --- |
| Age (years) | 52.4±17.1 (18-86) |
| Sex (male/female) | 84/135 |
| Education (years) | 13.1±3.7 (4-22) |
| Advanced CS scoring measures |  |
| Number of words | 93.4±30.4 (39-219) |
| Number of sentences | 18.2±5.2 (4-40) |
| Number of nouns* | 21±8.1 (6-45) |
| Number of verbs* | 21.2±6.1 (4-42) |
| Number of functions words* | 37.8±13.1 (13-94) |
| Nouns/words ratio | .22±.04 (.13-.35) |
| Verbs/words ratio | .23±.04 (.08-.33) |
| Function words/words ratio | .40±.04 (.27-.50) |
| Notes. CS=Connected Speech; HP=healthy participant. *measures not included within the actual scoring system, which are displayed for descriptive purposes only. | |
